# The Effects of Intravenous Dexmedetomidine Injections on IOP in General Anesthesia Intubation: A Meta-Analysis

**DOI:** 10.1155/2017/6186832

**Published:** 2017-02-02

**Authors:** Chengmao Zhou, Yu Zhu, Zhen Liu, Lin Ruan

**Affiliations:** ^1^Department of Anesthesiology, Affiliated Tumor Hospital of Guangxi Medical University, Nanning 530021, China; ^2^Zhaoqing Medical College, Zhaoqing 526000, China

## Abstract

*Objective*. The aim of this meta-analysis is to evaluate the effects of dexmedetomidine on intraocular pressure (IOP) in patients with general anesthesia administered via intubation.* Methods*. We searched randomized controlled trials (RCT) on the effects of intravenous injection of dexmedetomidine on IOP in patients with general anesthesia administered via intubation.* Results*. The meta-analysis study showed that (1) a statistically significant difference [WMD = −3.40 mmHg, 95% CI (−4.76, −2.04), *P* < 0.00001] was found between IOP of the two groups. (2) The IOP of the dexmedetomidine group that was administrated succinylcholine was lower than that of placebo group which was administrated succinylcholine [WMD = −4.13 mmHg, 95% CI (−6.01, −2.25), *P* < 0.0001]. (3) Compared with the IOP of patients in the placebo group, patients with intubation in the dexmedetomidine group maintained a lower IOP [WMD = −3.10 mmHg, 95% CI (−5.12, −1.07), *P* = 0.003]. However, for incidences of bradycardia, the use of dexmedetomidine was higher than that of the placebo [RR = 0.23, 95% CI (0.07, 0.76), *P* = 0.02].* Conclusion*. This meta-analysis showed that, in many cases, dexmedetomidine can lower the IOP of patients with general anesthesia administered by intubation.

## 1. Introduction

The induction of general anesthesia and endotracheal intubation have a significant impact on hemodynamics and the intraocular pressure (IOP) of patients during the induction period. In addition, patients with hypertension, coronary heart disease, or ophthalmological disease can also be affected. As a result, it is crucial for patients to get through the induction period of general anesthesia. Dexmedetomidine can diminish activity in the sympathetic nervous system via highly selective central nervous system and peripheral *α*2 adrenergic receptors, thus stabilizing hemodynamics [1, 2]. Studies have shown that dexmedetomidine is able to prevent the increase of IOP caused by cannula stimulation and succinylcholine. However, other studies have shown that dexmedetomidine has no influence on IOP in patients with general anesthesia administered by intubation [3, 4]. This meta-analysis was designed to investigate the influence of dexmedetomidine on IOP during the induction period of general anesthesia.

## 2. Materials and Methods

### 2.1. Inclusion and Exclusion Criteria

#### 2.1.1. Inclusion Criteria

Inclusion criteria were as follows: (1) types of studies: RCT (randomized control trial), (2) study subjects: patients with general anesthesia intravenously administered by dexmedetomidine injected via intubation, (3) interventions: dexmedetomidine and placebo, and (4) outcome measurement indicators: IOP.

#### 2.1.2. Exclusion Criteria

 Exclusion criteria were as follows: no full text or related data, no placebo control group, or an operation which was not standardized.

## 3. Search Strategy

We conducted our search using both a combination of subject terms and free words in PUBMED, The Cochrane Library, and CNKI. We searched for terms such as dexmedetomidine and intraocular pressure. The following search strategy was used: (“dexmedetomidine” [Mesh] AND “Intravenous” [Mesh] AND “General Anesthesia” [Mesh] AND (“Intraocular Pressure” [Mesh] OR “Ocular Hypertension” [Mesh]).

### 3.1. Search Process

**Figure 1:**
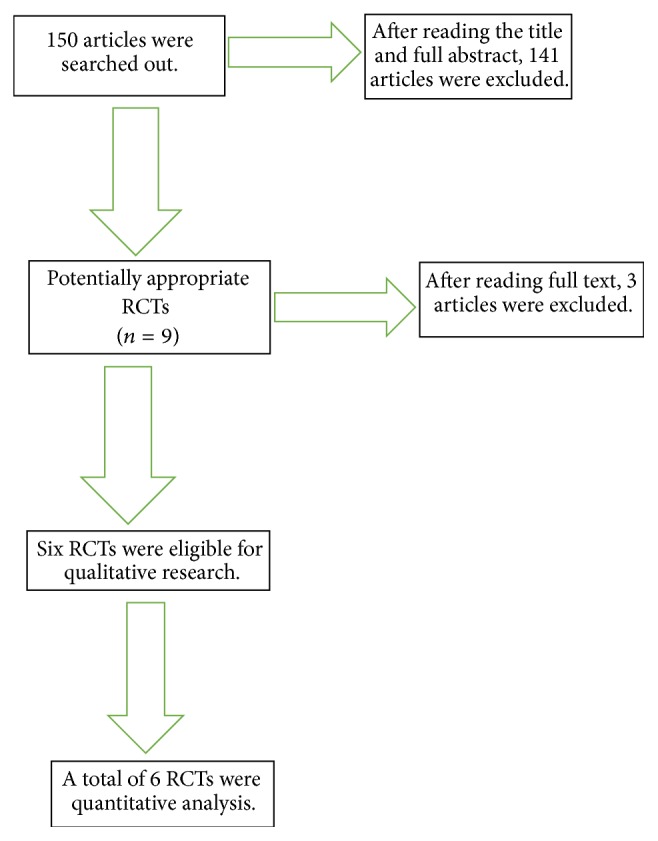
Flow diagram.

The detailed search process is shown in Figure 1.

### 3.2. Literature Screening and Data Extraction

Two evaluators independently read relevant articles and abstracts of related articles. A third evaluator then read the full text and assessed the quality of articles that had met the inclusion criteria. The two discussed or consulted a third party when there was a disagreement. We extracted relevant information and assessed quality of articles that were in accordance with the inclusion criteria. When met with multigrouped data, we merged them based on formulas of the Cochrane reviewers Manual Version 5.1.0.

The primary outcome was mean IOP with standard deviation (SD). The secondary outcomes were hypotension and bradycardia.

### 3.3. Quality of Articles and Literature Assessment

We evaluated the methodological quality of included studies according to risk of bias assessment tools from the Cochrane Handbook for Systematic Reviews of Interventions (Version, 5.1.0) and then adopted a modified Jadad scale for assessment. Evaluation contents included the following: (1) randomization; (2) description of withdrawals and drop outs; (3) blinding; (4) incomplete outcome indicators.

### 3.4. Statistical Analysis

We conducted the meta-analysis using RevMan 5.2 (Review Manager 5.2 is the software used for preparing and maintaining Cochrane Reviews) and employed weighted mean difference (WMD) and standardized mean difference (SMD) for continuous variables and 95% confidence intervals (95% CI) for statistical analysis. A heterogeneity test was done on included studies via an *χ*^2^ and *I*^2^ test. When *P* ≥ 0.05 and *I*^2^ ≤ 50%, indicating that statistical heterogeneity existed among the studies, a fixed-effects model was used. A random-effects model was employed when *P* < 0.05 and *I*^2^ > 50%, suggesting that heterogeneity could be found among these studies. We used a funnel plot to assess publication bias.

## 4. Results

### 4.1. Literature Search Results

At first, nine articles were included in the study, based on the inclusion criteria. Three of them [5–7] were excluded after a full-text reading because they lacked relevant data. In total, 6 articles [3, 4, 8–11] were included in the study.

### 4.2. Characteristics of the Included Studies and Methodological Quality Assessment

Six articles totaling 324 patients were included in this meta-analysis. All included studies covered research using RCTs. All provided the details of their blinding methods. The Jadad score is provided in Table 1.

#### 4.2.1. IOP of Patients Premedicated with Dexmedetomidine or Placebo

**Figure 2:**
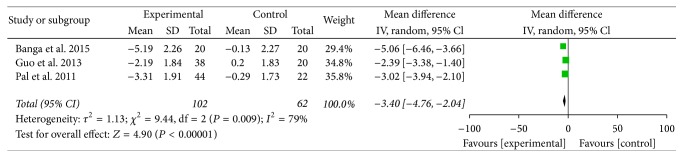
IOP of patients premedicated with either dexmedetomidine or the placebo.

Three studies totaling 164 patients were included in our meta-analysis. The IOPs of all three studies were compared with those of patients who had been administrated suxamethonium. Statistical heterogeneity (*I*^2^ = 79%) was found among the three studies; thus a random-effects model was employed to conduct the meta-analysis. The results showed that there was statistical significance [WMD = −3.40 mmHg, 95% CI (−4.76, −2.04), *P* < 0.00001] of IOP between the two groups and that the IOP of dexmedetomidine was lower than that of the control group (see Figure 2). Further subgroup analyses according to different surgeries and doses of dexmedetomidine did not affect the pooled results, and all of these analyses were also influenced by heterogeneity.

#### 4.2.2. IOP of Patients Administrated Suxamethonium

**Figure 3:**
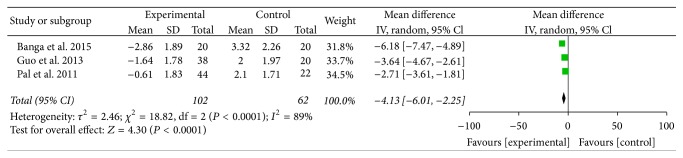
IOP of patients administrated suxamethonium.

Three studies totaling 164 patients were included. The IOPs from all three studies were compared with those of patients who had been administrated suxamethonium. Statistical heterogeneity (*I*^2^ = 89%) was found among the three studies, and thus a random-effects model was used to conduct the meta-analysis. The results suggested that there was statistical significance [WMD = −4.13 mmHg, 95% CI (−6.01, −2.25), *P* < 0.0001] and that the IOP of dexmedetomidine was lower than that of the control group (see Figure 3). Further subgroup analyses according to different surgeries and doses of dexmedetomidine did not affect the pooled results, and all of these analyses were also influenced by heterogeneity.

#### 4.2.3. IOP of Patients with Intubation

**Figure 4:**
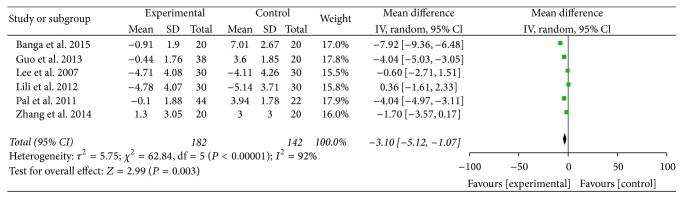
IOP of patients with intubation.

Six studies totaling 324 patients were included. The IOPs from all six studies were compared with those of patients with intubation. Statistical heterogeneity (*I*^2^ = 92%) was found among the six studies; thus a random-effects model was adopted for the meta-analysis. The results suggested that there was statistical significance [WMD = −3.10 mmHg, 95% CI (−5.12, −1.07), *P* = 0.003] and that the IOP of dexmedetomidine was lower than that of the control group (see Figure 4). Further subgroup analyses according to different surgeries and doses of dexmedetomidine did not affect the pooled results, and all of these analyses were also influenced by heterogeneity.

#### 4.2.4. Dexmedetomidine versus Placebo for Cardiovascular Events Control

**Figure 5:**
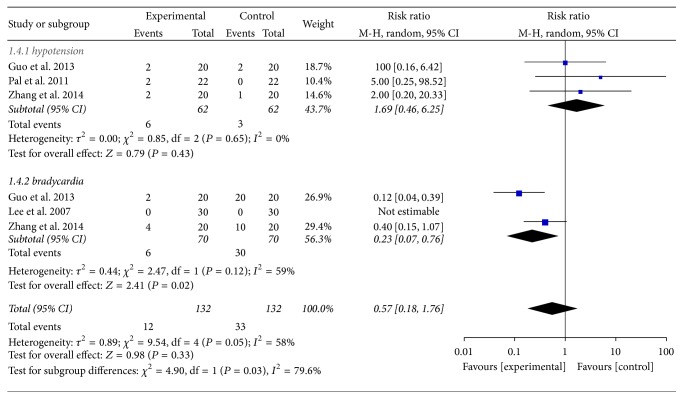
Dexmedetomidine versus placebo for the cardiovascular events control.

This meta-analysis showed that there was no significant difference in the incidence of the use of dexmedetomidine for hypotension when compared with the placebo (RR = 1.69, 95% CI (0.46, 6.25), *P* = 0.43). There was no statistical evidence of heterogeneity among the studies (*I*^2^ = 0; *P* = 0.65). However, there was a significantly higher incidence of treatment for bradycardia (RR = 0.23, 95% CI (0.07, 0.76), *P* = 0.02) without statistical evidence of heterogeneity among the studies (*I*^2^ = 59%; *P* = 0.12). There was a significant heterogeneities, and therefore a random-effects model was used (see Figure 5). Further subgroup analyses according to different surgeries and doses of dexmedetomidine did not affect the pooled results, and all of these analyses were also influenced by heterogeneity.

#### 4.2.5. The Funnel Plot Indicates That the Results Were Not Asymmetrical

**Figure 6:**
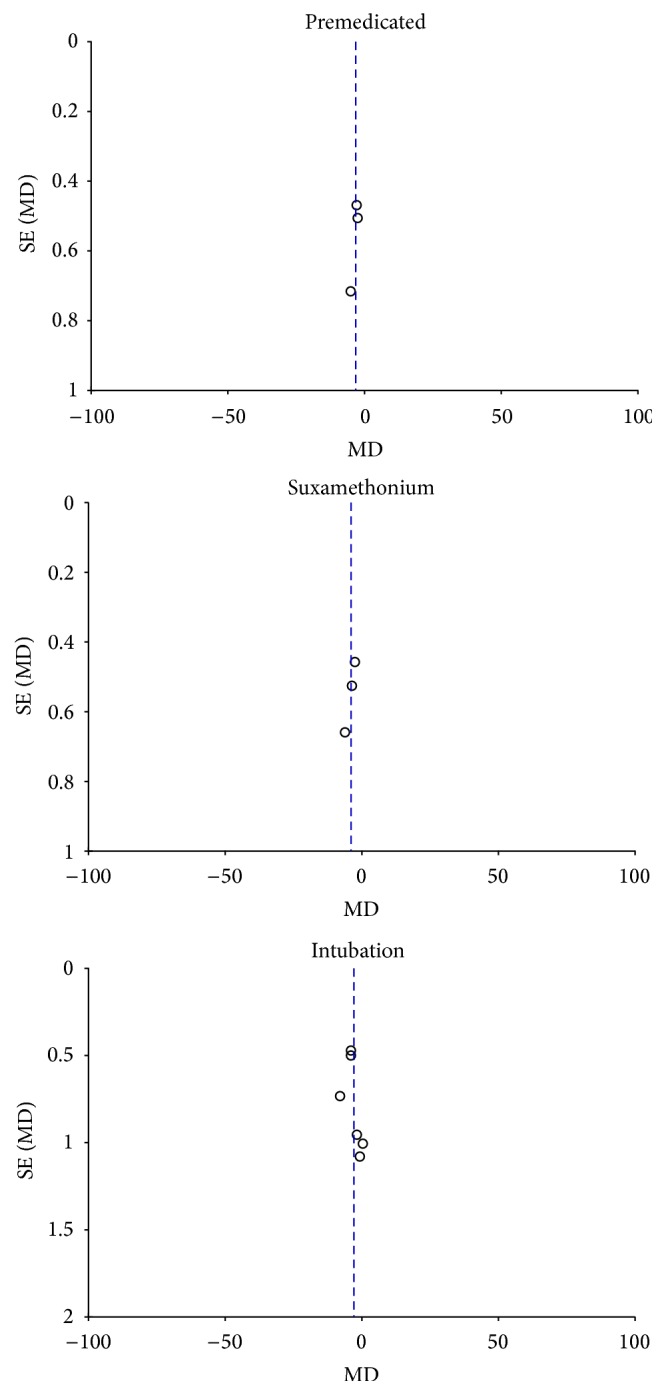
Funnel plot.

There was also no publication bias (see Figure 6).

## 5. Discussion

The meta-analysis showed that dexmedetomidine can reduce IOP elevation in patients with general anesthesia administered by intubation. This is consistent with the three RCTs [5–7] excluded from the study. However, the meta-analysis also suggested an increased risk of bradycardia after use of dexmedetomidine, compared with the placebo.

IOP refers to the contents of the eye wall of the pressure acting on the eye. Elevation of IOP oppresses arteria centralis retinae and decreases ocular perfusion pressure. This can lead to retinal ischemia and, in severe cases, results in obstruction of the central retinal artery, thus causing visual impairment. IOP normally ranges from 10 to 22 mmHg. It is essential to control perioperative IOP because IOP elevation may cause temporary blindness or induce acute glaucoma.

Moreover, patients experiencing ocular trauma during anesthesia are often those who go into surgery without an empty stomach. When this occurs, it is essential that an airway (endotracheal intubation) is quickly established without increasing IOP or aspiration. After administrating nondepolarizing neuromuscular blocking drugs for 60–90 seconds, an ideal window for endotracheal intubation will quickly emerge. Succinylcholine is still one of the most common muscle relaxants used for rapid endotracheal intubation; however it elevates IOP.

Dexmedetomidine, as a new *α*2 adrenergic receptor agonist, has its own pharmacological properties of sedation, analgesia, inhibition of anxiety, and autonomic reflexes. It also causes no respiratory depression and can inhibit the release of norepinephrine and diminish the incidence of hypertension inflicted by a variety of stimuli during surgery. Dexmedetomidine may reduce IOP via direct contraction of ball arteries in the ciliary body and thus reduce aqueous humor and angiotasis of the aqueous discharge system, thereby increasing the discharge of the aqueous humor [9, 12]. The reason for the reduction in IOP may be the hemodynamic effect of dexmedetomidine.

This meta-analysis has its limitations, listed as follows. Firstly, a small number of studies was included in this meta-analysis, affecting the explanatory power of its claims. In addition, considering the uniqueness of intraocular injection drug patients, injection drug concentration, frequency, baseline IOP, and many other factors, it is necessary to adopt a more rigorous research design and to improve system quality level of proof so as to perfect the sustainability of true assessment. However, we believe that dexmedetomidine does reduce patients' IOP and mitigates the elevation of IOP in patients with intubation. This is achieved by administrating succinylcholine. However, there are data to support the risk of bradycardia when using dexmedetomidine in a clinical setting.

## Figures and Tables

**Table 1:** Characteristics of the included studies in the meta-analysis.

Author (year of publication)	Headcount	Grouping	Surgical setting	Jadad score
Lee et al. 2007 [4]	60	Dexmedetomidine	Elective vitreoretinal surgery	6
		Normal saline		

Pal et al. 2011 [8]	66	Dexmedetomidine	Elective nonophthalmic surgery	5
		Normal saline		

Banga et al. 2015 [9]	60	Dexmedetomidine	Elective nonophthalmic surgery	5
		Normal saline		
		Clonidine		

Lili et al. 2012 [3]	60	Dexmedetomidine	Vitreoretinal surgery	6
		Normal saline		

Guo et al. 2013 [10]	60	Dexmedetomidine	Elective nonophthalmic surgery	4
		Normal saline		

Zhang et al. 2014 [11]	40	Dexmedetomidine	Gynecologic surgery	4
		Normal saline		

## References

[1] Carollo D. S., Nossaman B. D., Ramadhyani U. (2008). Dexmedetomidine: a review of clinical applications. *Current Opinion in Anaesthesiology*.

[2] Bylund D. B., Blaxall H. S., Iversen L. J. (1992). Pharmacological characteristics of alpha 2-adrenergic receptors: comparison of pharmacologically defined subtypes with subtypes identified by molecular cloning. *Molecular Pharmacology*.

[3] Lili X., Jianjun S., Haiyan Z. (2012). The application of dexmedetomidine in children undergoing vitreoretinal surgery. *Journal of Anesthesia*.

[4] Lee Y. Y. S., Wong S. M., Hung C. T. (2007). Dexmedetomidine infusion as a supplement to isoflurane anaesthesia for vitreoretinal surgery. *British Journal of Anaesthesia*.

[5] Kim N. Y., Yoo Y.-C., Park H., Choi Y. D., Kim C. Y., Bai S. J. (2015). The effect of dexmedetomidine on intraocular pressure increase in patients during robot-assisted laparoscopic radical prostatectomy in the steep trendelenburg position. *Journal of Endourology*.

[6] Mowafi H. A., Aldossary N., Ismail S. A., Alqahtani J. (2008). Effect of dexmedetomidine premedication on the intraocular pressure changes after succinylcholine and intubation. *British Journal of Anaesthesia*.

[7] Jaakola M.-L., Ali-Melkkila T., Kanto J., Kallio A., Scheinin H., Scheinin M. (1992). Dexmedetomidine reduces intraocular pressure, intubation responses and anaesthetic requirements in patients undergoing ophthalmic surgery. *British Journal of Anaesthesia*.

[8] Pal C. K., Ray M., Sen A., Hajra B., Mukherjee D., Ghanta A. K. (2011). Changes in intraocular pressure following administration of suxamethonium and endotracheal intubation: influence of dexmedetomidine premedication. *Indian Journal of Anaesthesia*.

[9] Banga P. K., Singh D. K., Dadu S., Singh M. (2015). A comparative evaluation of the effect of intravenous dexmedetomidine and clonidine on intraocular pressure after suxamethonium and intubation. *Saudi Journal of Anaesthesia*.

[10] Guo W., Zhang Z. P., Fang N. N. (2013). Effects of different dosage of dextomidine on the intraocular pressure changes after succinylcholine administration and endotracheal intubation. *Journal of Clinical Anesthesiology*.

[11] Zhang L., Li X., Chen Q. (2014). Effect of dexmedetomidine on perioperative blood dynamics and intraocular pressure undergoing general anesthesia gynecologic laparoscopic surgery. *The Chinese Journal of Clinical Pharmacology*.

[12] Vartiainen J., MacDonald E., Urtti A., Rouhiainen H., Virtanen R. (1992). Dexmedetomidine-induced ocular hypotension in rabbits with normal or elevated intraocular pressures. *Investigative Ophthalmology & Visual Science*.

